# Use of an immobilised thermostable *α*-CA (SspCA) for enhancing the metabolic efficiency of the freshwater green microalga *Chlorella sorokiniana*

**DOI:** 10.1080/14756366.2020.1746785

**Published:** 2020-03-30

**Authors:** Giovanna Salbitani, Sonia Del Prete, Francesco Bolinesi, Olga Mangoni, Viviana De Luca, Vincenzo Carginale, William A. Donald, Claudiu T. Supuran, Simona Carfagna, Clemente Capasso

**Affiliations:** a Department of Biology, University of Naples Federico II, Napoli, Italy; b Department of Biology, Agriculture and Food Sciences, CNR, Institute of Biosciences and Bioresources, Napoli, Italy; c School of Chemistry, University of New South Wales, Sydney, Australia; d Department of NEUROFARB, Section of Pharmaceutical and Nutraceutical Sciences, University of Florence, Polo Scientifico, Firenze, Italy

**Keywords:** *Chlorella sorokiniana*, carbonic anhydrase, thermostable SspCA, photosynthetic efficiency, carotenoids production, hydratase activity

## Abstract

There is significant interest in increasing the microalgal efficiency for producing high-quality products that are commonly used as food additives in nutraceuticals. Some natural substances that can be extracted from algae include lipids, carbohydrates, proteins, carotenoids, long-chain polyunsaturated fatty acids, and vitamins. Generally, microalgal photoautotrophic growth can be maximised by optimising CO_2_ biofixation, and by adding sodium bicarbonate and specific bacteria to the microalgal culture. Recently, to enhance CO_2_ biofixation, a thermostable carbonic anhydrase (SspCA) encoded by the genome of the bacterium *Sulfurihydrogenibium yellowstonense* has been heterologously expressed and immobilised on the surfaces of bacteria. Carbonic anhydrases (CAs, EC 4.2.1.1) are ubiquitous metalloenzymes, which catalyse the physiologically reversible reaction of carbon dioxide hydration to bicarbonate and protons: CO_2_ + H_2_O ⇄ HCO_3_^−^ + H^+^. Herein, we demonstrate for the first time that the fragments of bacterial membranes containing immobilised SspCA (M-SspCA) on their surfaces can be doped into the microalgal culture of the green unicellular alga, *Chlorella sorokiniana*, to significantly enhance the biomass, photosynthetic activity, carotenoids production, and CA activity by this alga. These results are of biotechnological interest because *C. sorokiniana* is widely used in many different areas, including photosynthesis research, human pharmaceutical production, aquaculture-based food production, and wastewater treatment.

## 1. Introduction

Photosynthesis employs sunlight and the reaction between CO_2_ and H_2_O to generate carbohydrates and oxygen as a side product. This gas is necessary for the aerobic respiration but also promotes the formation of the ozone layer in the upper atmosphere. During the photosynthetic reactions, the energy of sunlight is converted into chemical energy, i.e., ATP and NADPH, which are thereafter involved in the biosynthesis of carbohydrates from CO_2_ as a unique carbon source^1^^,^^2^. The aerobic respiration (glucose + O_2_ → H_2_O + CO_2_), on the contrary, is the process of energy production, which converts sugars into carbon dioxide and water. These two opposite reactions influence the global carbon cycle, being fundamental for most life forms on earth^2^. The light-dependent reactions to form glucose and other carbohydrates are known as the Calvin-Benson cycle. There are three photosynthetic pathways, C_3_, C_4_, and CAM (Crassulacean Acid Metabolism) that exist among terrestrial plants^3^^,^^4^. In the C_3_ photosynthesis, which is the most ancestral form, the enzyme ribulose bisphosphate carboxylase-oxygenase (RuBisCO)^5^, which is present in the chloroplast stroma of C_3_ plants, combines the ribulose-1,5-bisphosphate (RuBP), a molecule containing five carbon atoms, with CO_2_ to form two molecules of phosphoglycerate (PGA, a 3-carbon molecule)^6^^,^^7^. In the C_4_ pathway, the CO_2_ is converted into bicarbonate, which is subsequently reacted with phosphoenolpyruvic acid (PEP), a 3-carbon molecule, in the presence of phosphoenolpyruvate carboxylase (PEPC)^8^. The product of this reaction is a 4-carbon molecule, oxaloacetic acid (OAA), which is thereafter reduced to malate, another four-carbon acid^8^. The CAM pathway was documented for the first time in plant families that are adapted to very arid regions, such as many epiphytes and succulents^9^. These plants have a dual pathway of carboxylation temporally separated into the same tissue. In the night with the stomata opening, the CO_2_ is fixed as an organic acid form of the anion malate by PEPC. In contrast, during the day, with the stomatal closure, the malic acid undergoes decarboxylation, determining an increase of CO_2_ around the enzyme RuBisCO of about 60 times the ambient levels, allowing the photosynthetic reaction typical of the C3 cycle mentioned above^9^. The RuBisCO enzyme also uses O_2_ as substrate, not only CO_2_^10^_._ The rate of the oxygenation and carboxylation by RuBisCO is controlled by the levels of O_2_ and CO_2_ and is the primary factor in determining the efficiency of the photosynthetic process^11^. CAM is one example of a carbon-concentrating mechanism (CCM) in higher order plants, in which, as mentioned above, decarboxylation of malic acids affords supplementary amounts of CO_2_.

Microalgal growth is driven by the same photosynthetic process present in higher plants^12–15^. Both freshwater and marine microalgae, also developed a CCM to increase CO_2_ concentration close to that for RuBisCO that is up to 1000-fold compared to the low CO_2_ concentrations found in aquatic environments^16^. In the single-cell green alga, *Chlamydomonas reinhardtii,* the microalgal inorganic carbon uptake has been well described^17^^,^^18^. It involves the diffusion of CO_2_ and transport of HCO_3_^−^ across the microalgal membranes and the interconversion of CO_2_ and HCO_3_^−^ by the algal carbonic anhydrases (CAs, EC 4.2.1.1), with the final result of concentrating the CO_2_ in the proximity of RuBisCO^17^, which is localised mostly within the pyrenoids, the chloroplast microcompartments found in algae^19–23^. In cyanobacteria, carboxysomes are the equivalent of the pyrenoids^24–27^.

Indeed, CAs are a superfamily of metalloenzymes, which catalyse the simple but physiologically reversible and crucial reaction of carbon dioxide hydration to bicarbonate and protons: CO_2_ + H_2_O ⇄ HCO_3_^−^ + H^+^^28–35^. To date, CAs are categorised into eight genetically distinct families (or classes), named with the Greek letters: α, β, γ, δ, ζ, η, θ, and ι^36^. The last three classes were only recently discovered^37–41^. The distribution of CA-classes is very variegated in most living organisms investigated so far. CAs present in animals belong to α-class^21^^,^^42^, plants and algae have α, β, γ, δ, ζ, θ and ι-classes; fungi encode for α and β-CAs; protozoa for α, β and/or η-CAs; bacteria for α, β, γ, and, as recently reported, for ι-CA classes^34^^,^^37^^,^^40^^,^^43–47^. The proposed physiological role of CAs in all these organisms is to regulate pH and to assist the transport of carbon dioxide and bicarbonate, making possible their balance inside the cells, which will not be ensured by the very low k_cat_ (0.15 s^−1^) of the uncatalyzed CO_2_ hydration/dehydration reaction^43^^,^^48–52^. All these roles of CAs have in the end crucial physiological functions for the metabolism of the organisms in which they are found^43^^,^^48–52^.

Recently, considerable and diverse efforts have been made to improve the efficiency of microalgal cultures, as they provide biomass abundant in high-value products, such as lipids, carbohydrates, and proteins^53^^,^^54^. Moreover, they are also a biological factory of carotenoids, long-chain polyunsaturated fatty acids, and vitamins, which are commonly used as food additives in nutraceuticals^53^^,^^55^^,^^56^. Generally, for maximising the microalgal biomass during the photoautotrophic growth, the microalgal cultures are usually optimised improving the CO_2_-fixation or, by adding sodium bicarbonate^57^^,^^58^ or specific bacteria^59^. Recently, our groups heterologously expressed and immobilised on the surface of bacterial hosts a thermostable *α*-CA (SspCA is the acronym) from the bacterium *Sulfurihydrogenibium yellowstonense*^60^. This approach, entitled *in vivo* immobilisation, was achieved by transforming the *E. coli* cells with a plasmid containing a chimeric gene resulted by the fusion of a signal peptide (pelB gene), which directs the neosynthesized protein to the bacterial periplasmic space; the gene (INPN gene) encoding for the *Pseudomonas syringae* INP domain, which anchors the neosynthesized protein to the bacterial outer membrane (external side); and the gene encoding for the thermostable enzyme SspCA^60^. The anchored SspCA was thus efficiently overexpressed on the external bacterial surface of *E. coli* and was stable and active for 15 h at 70 °C and for many days at 25 °C^60^. Assuming that the CA activity facilitates the rapid conversion of the aqueous CO_2_ to HCO_3_^−^, we hypothesised that the addition of an exogenous and thermostable CA into the microalgal culture might enhance the algal bicarbonate uptake ameliorating the microalgal growth. Thus, in the present paper, this concept was investigated for the first time and used to enhance the biomass, photosynthetic activity, carotenoids production, and CA activity of *Chlorella sorokiniana*. This was achieved by supplementing the microalgal culture with fragments of bacterial membranes containing the immobilised SspCA on their surface and biocarbonate. Furthermore, the results were compared with those obtained by adding only sodium bicarbonate at the concentration of 1.0 *g*/L.

## 2. Materials and methods

### 2.1. Chemicals and instruments

All the chemicals used in this study were of reagent grade and purchased from Sigma and GE Healthcare. SDS–PAGE apparatus was procured by BioRAD.

### 2.2. Protein determination

The protein quantification was carried out by Bradford method (BioRAD)^61^.

### 2.3. Preparation of the bacterial membrane with the immobilised SspCA

Competent *E. coli* BL21 (DE3) cells were transformed with construct indicated with the acronym pET-22b/INPN-SspCA and prepared as describe by Del Prete et al.^60^. Bacterial cells were grown at 37 °C, and when cells reached an OD_600_ of 0.6–0.7, the protein surface expression was induced with 0.5 mM isopropyl-thio-b-D-galactoside (IPTG) and 0.5 mM ZnSO_4_. After additional growth for 6 h, the cells were harvested by centrifugation and washed three times with PBS. Aliquots of cells were resuspended in 25 mM Tris-HCl, pH 8.0. Membrane fragments containing the immobilised SspCA (M-SspCA) were prepared disrupting the cells by sonication (10 s, for 10 cycles). 0.5 g of M-SspCA were added at time 0 and 48 h to the algal medium containing the bicarbonate.

### 2.4. Assay for carbonic anhydrase using CO_2_ as substrate

CA activity assay was performed as described by Capasso et al.^62^. Briefly, the assay was based on the monitoring of pH variation due to the catalysed conversion of CO_2_ to bicarbonate. Bromothymol blue was used as the indicator of pH variation and the assay was performed at 0 °C. The CO_2_-satured solution was used as substrate. To test the activity of carbonic anhydrase, 1.0 mL of 25 mM Tris, pH 8.3, containing bromothymol blue as a dye (to give a distinct and visible blue colour) was added to two test tubes chilled in an ice bath. An appropriate amount of the enzyme solution (e.g. microalgal cell extract) were added to one tube, and an equivalent amount of buffer was added to the second tube as control. One millilitre of CO_2_ solution was added, and the time required for the solution to change from blue to yellow was recorded (transition point of bromothymol blue is pH 6.0–7.6). The time required for the colour change is inversely related to the quantity of enzyme present in the sample. Wilbur-Anderson units were calculated according to the following definition: One Wilbur-Anderson unit (WAU) of activity is defined as (T_0_−T)/T, where T_0_ (uncatalyzed reaction) and T (catalysed reaction) are recorded as the time (in seconds) required for the pH to drop from 8.3 to the transition point of the dye in a control buffer and in the presence of enzyme, respectively.

### 2.5. Protonography

To prepare *Chlorella* crude extract for protonography, aliquots of 200 mL of algal culture were harvested by centrifugation at 4000 *g* for 7 min; the pellets were re-suspended in 4.0 mL of cold extraction buffer (50 mM Tris-HCl, pH 8.3) and the cells were lysed by two passage at 1100 psi through French pressure cell (Aminco). The lysate was cleared by centrifugation at 12,000 *g* for 30 min at 4 °C and the obtained supernatant represented the crude extract. To perform the protonography, wells of 12% SDS-PAGE gel prepared as described by Laemmli^63^, were loaded with an appropriate amount of the microalgal crude extract mixed with loading buffer without 2-mercaptoethanol and without boiling the samples, in order to avoid protein denaturation. The gel was run at 150 V until the dye front ran off the gel. Following the electrophoresis, the 12% SDS-PAGE gel was treated as described by Capasso et al.^64–67^ to detect the yellows bands due to the hydratase activity.

### 2.6. Algal strains and growth conditions

*Chlorella sorokiniana* Shihira and Krauss, strain 211/8k (CCAP of Cambridge University) was grown in Erlenmeyer flask at 30 ± 2.5 °C, under continuously light (led panel, 70 µmol photons m^−2^ s^−1^) and flushed with air. The composition of the basal medium was previously reported by Salbitani et al.^68^, and the pH of the medium was 7.5 at T_0_. Cultures of *C. sorokiniana* were grown with a supplementation of 0 (control) and 1.0 g L^−1^ of NaHCO_3_. The bicarbonate was added, in a single administration at T_0_, when cultures were into lag phase (∼2.0 × 10^6^ cell mL^−1^). To some cultures supplemented with bicarbonate 1.0 g L^−1^, at T_0_ or T_2_ fragments of bacterial membranes (0.5 g) with the immobilised thermostable SspCA were added. The number and cells size of *Chlorella* were determined by Countess II FL Automated Cell Counter (Thermo Fisher Scientific) equipped with a fluorescence filter (Ex 628/40, Em 692/40; EVOS Light Cube for Cy5; Termo Fisher Scientifc Inc.).

### 2.7. Pigment contents

Total chlorophyll and carotenoids contents were estimated spectrophotometrically after extraction into N,N-dimethylformamide according to Inskeep and Bloom^69^ and Wellburn^70^, respectively.

### 2.8. Photosynthetic efficiency

The maximum PSII photochemical efficiency (F_v_/F_m_) has been determined using a Phyto_PAM II compact unit (Walz). All samples were acclimated at the dark for 30 min before the analysis to minimise the non-photochemical dissipation of excitation, and measurements were blank corrected filtering the sample through 0.2 μm filter^71^. As regard F_v_/F_m_, samples were illuminated with a saturating pulse following as reported in Maxwell and Jonson^72^, and values derived from the formula F_v_/F_m_ = (F_m_−F_0_)/F_m_.

## 3. Results and discussion

### 3.1. Basal hydratase activity of the endogenous microalgal CAs

The analysis of the genome belonging to different microalgal species evidences a very variegated pattern of CA classes. It is possible to identify seven of the eight CA-classes discovered up to now in these organisms. The different classes can coexist or have different localizations inside the cells, such as the cell wall, plasma membrane, cytosol, mitochondria, chloroplast stroma, and chloroplast thylakoid lumen^73–75^. Besides, for each enzyme class, many isoforms were reported to exist^74^. In the present manuscript, the interest was focussed on the freshwater green microalga *Chlorella sorokiniana* as it can be useful in many fields, such as photosynthesis research, pharmaceuticals for humans, aquaculture foods, and wastewater treatment. In 1998, a soluble form of CA belonging to the *α*-class^76^ was purified and characterised from *C. sorokiniana*. Other CA-classes appear to be encoded by this green microalga genome although they have not yet been characterised^76^.

**Figure 1.**
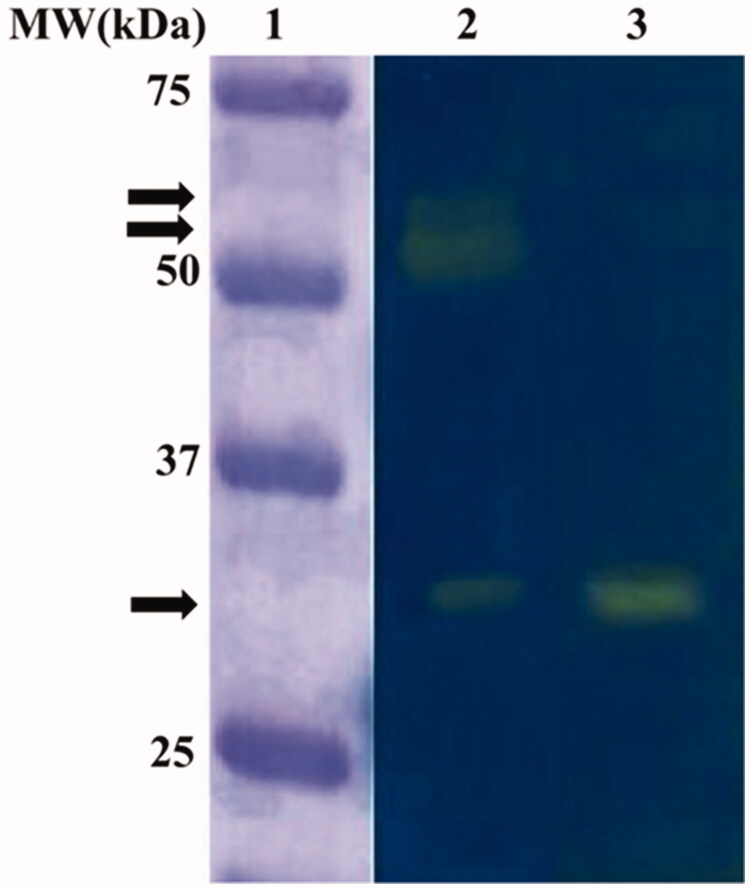
Microalgal endogenous activity revealed by the protonography analysis. Legend: Lane 1, molecular markers; Lane 2, *C. sorokiniana* cellular extract; Lane 3, commercial bovine CA used as positive controls. The arrows identified the yellow bands corresponding to CO2 hydratase activity due to the microalgal CAs.

The *C. sorokiniana* extract was subjected to the Wilbur-Anderson (WA) assay and protonography, to investigate the microalgal endogenous CA hydrates activity. Using CO_2_ as a substrate, the CA specific activities of the microalgal extract resulted to be 20 ± 0.7 WAU/mg. The protonography analysis, which is specific for the detection of the CO_2_ hydratase activity on the polyacrylamide gel, was performed treating the SDS-PAGE gel with blue bromothymol, which is blue in its deprotonated form. The production of H^+^ ions, due to the CA hydratase activity, lowers the pH of the solution to pH 6.8, the colour transition point of the dye, developing a yellow band in correspondence of the hydratase activity. As a positive control, the commercial bovine *α*-CA (bCA) has been used. Figure 1 shows the protonogram obtained using the *C. sorokiniana* crude extract. Intriguing, the protonogram evidenced three yellow bands corresponding to the CO_2_ hydratase activity (Figure 1). One band is at the gel position corresponding to 29 kDa, the molecular weight of the *C. sorokiniana* *α*-CA, whereas the other two bands (very close to each other) are visible at a molecular weight above 50 kDa. The latter two bands could be a different oligomeric state of the *α*-CA (not wholly dissociated in the monomer form with MW of about 29 kDa) or a different class of the microalgal CAs not yet characterised (Figure 1). It is interesting to note that, these microalgal CAs, such as the CAs identified in other species^64^, can refold and generate their active form correctly after the removal of the SDS by the gel to accomplish the protonography analysis.

### 3.2. Microalgal growth

*C. sorokiniana* cells were cultivated under four different conditions: (1) Control: culture growth in the basal medium; (2) Bic: culture supplemented at T_0_ (0 h) with bicarbonate at 1.0 g L^−1^; (3) M-SspCA-0: culture supplemented at T_0_ with bicarbonate (1.0 g L^−1^) and fragments of bacterial membranes (0.5 g) with the immobilised thermostable SspCA; (4) M-SspCA-2: culture supplemented at T_0_ with bicarbonate (1.0 g L^−1^) and at T_2_ (48 h) with fragments of bacterial membranes (0.5 g) having the immobilised thermostable SspCA.

**Figure 2.**
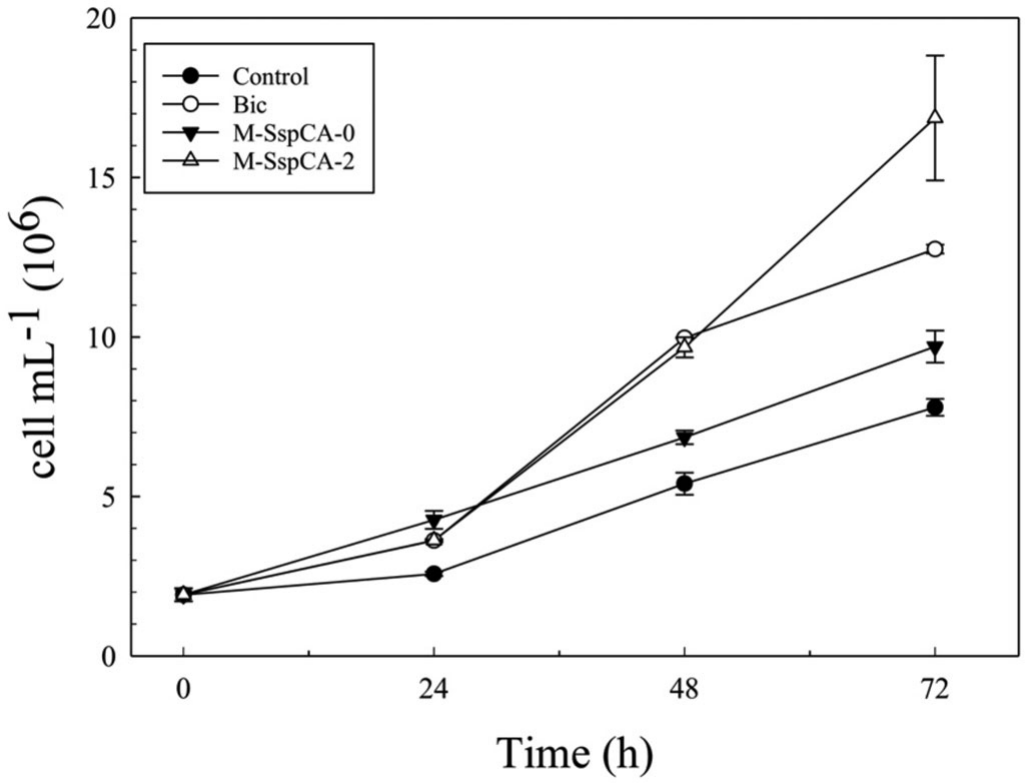
Cellular density (cells mL^−1^) in control and experimental cultures (Bic, M-SspCA-0, M-SspCA-2) of *Chlorella sorokiniana*. Error bars represent SD (*n* = 3).

Figure 2 indicates the growth of *C. sorokiniana* under the four different conditions (specified above). The growth was monitored by determining the number of microalgal cells and the optical density (OD_800 nm_) of the cultures. The algae biomass growth based on the optical density was not shown because it displayed a profile very similar to that of the number of cells mL^−1^. From Figure 2 is readily apparent that when the microalgal medium was supplemented with bicarbonate or bicarbonate and M-SspCA at the two growing times (0 and 48 h), there was an increase, already at 24 h, in the number of cells with respect to the control (Figure 2). Interestingly, after 48 h, bicarbonate supplemented medium provoked an increase of the number of cells (9.97 × 10^6^ ± 0.015 cell mL^−1^) that was similar to that for M-SspCA-2, and 1.5 times higher compared to the number of cells (6.85 × 10^6^ ± 0.212 cell mL^−1^) obtained using bicarbonate plus M-SspCA added at the initial time. Obviously, up to 48 h the Bic and M-SspCA-0 cultures proceed in a completely similar manner. Intriguing, the addition of M-SspCA at 48 h (M-SspCA-2), resulted in better microalgal growth, generating, at 72 h, an evident increase in the number of the cells, which was of about 2.2 times (16.86 × 10^6^ ± 1.95 cell mL^−1^) than the control (7.8 × 10^6^ ± 0.26 cell mL^−1^), 1.7 time than M-SspCA-0 (9.7 × 10^6^ ± 0.5 cell mL^−1^) and 1.3 times than Bic (12.75 × 10^6^ ± 0.14 cell mL^−1^). We can speculate that the addition of the M-SspCA at the initial time transforms the bicarbonate in CO_2_, reducing the availability of bicarbonate and the uptake from the microalgal cells. On the contrary, the addition of M-SspCA at 48 h increased the number of cells mL^−1^. These can be explained considering that during the first 48 h, the microalgal growth in M-SspCA-2 cultures is supported only by bicarbonate; in fact, in absence of exogenous carbon dioxide, in M-SspCA-2 cultures, the bicarbonate is only in a very small part spontaneously converted in CO_2_. In addition, the bicarbonate is consumed by the algal growth and there is an accumulation of CO_2_; the addition of M-SspCA at 48 h immediately converts the CO_2_, from air and cellular metabolism, into bicarbonate, giving a further boost to microalgal growth. Thus, the bicarbonate coming from the catalysed reaction of the thermostable SspCA ameliorate the microalgal growth, as demonstrated by the fact that the number of cells is increased up to 17 × 10^6^ cell mL^−1^ (see Figure 2). This is corroborated by the fact that, as described in the literature, microalgal growth is improved by the addition of bicarbonate, reducing the microalgal oxidative stress induced by the macronutrient deficient conditions^77^^,^^78^.

**Figure 3.**
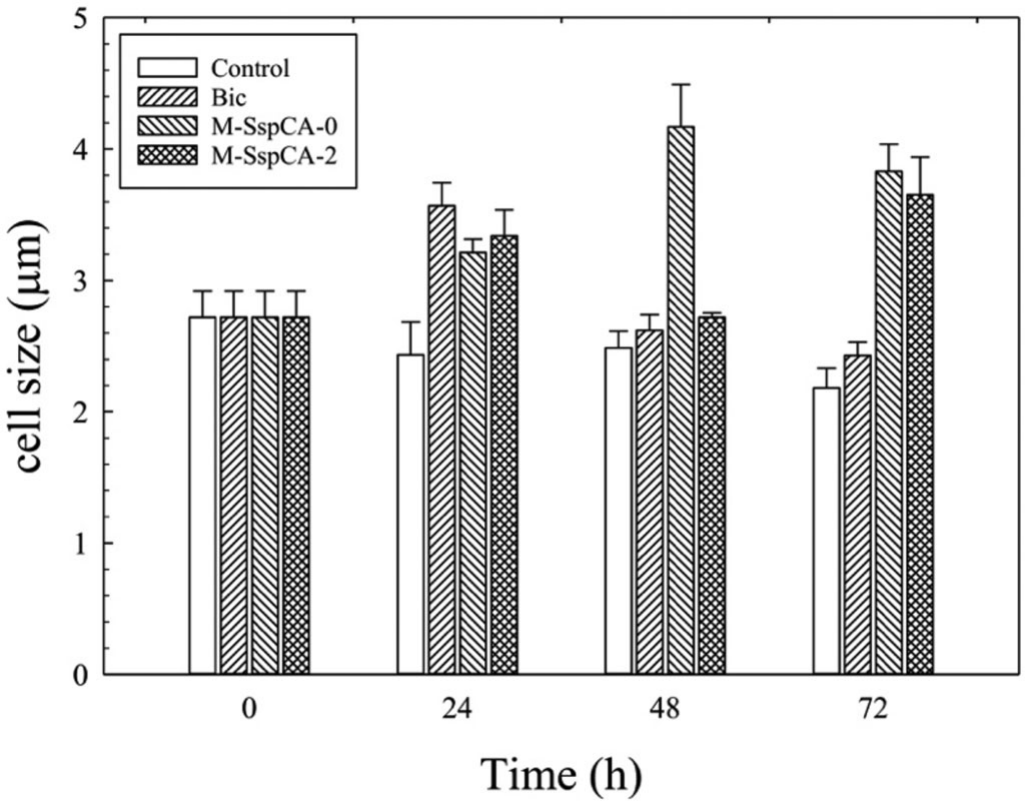
Average cell diameter of *C. sorokiniana* cells in control and experimental cultures (Bic, M-SspCA-0, M-SspCA-2). Error bars represent SD (*n* = 3).

Another exciting aspect is the difference in the *C. sorokiniana* cell size when the alga is grown in the four different conditions aforementioned. Figure 3 shows the microalgal cell size monitored at different times. As a result, at 24 h the cell size increased in the cultures supplemented with bicarbonate or bicarbonate plus the M-SspCA (added at 0 or 48 h) (Figure 3). It is fascinating to note that the addition of M-SspCA at 0 and 48 h determines at 72 h an increase of about 1.7 times of the microalgal cell size compared to the control. On the contrary, the medium supplemented with bicarbonate alone determined at 24 h an increase of the microalgal cell size of 1.3 times respect to the control, which slightly started to decrease after the 24 h (Figure 3). The increase of average cell diameter could be related to intracellular carbon storage; in fact, according to previous studies, bicarbonate supplementation to microalgae cultures promotes lipid accumulation^79^^,^^80^.

### 3.3. Microalgal photosynthetic efficiency (F_v_/F_m_)

**Figure 4.**
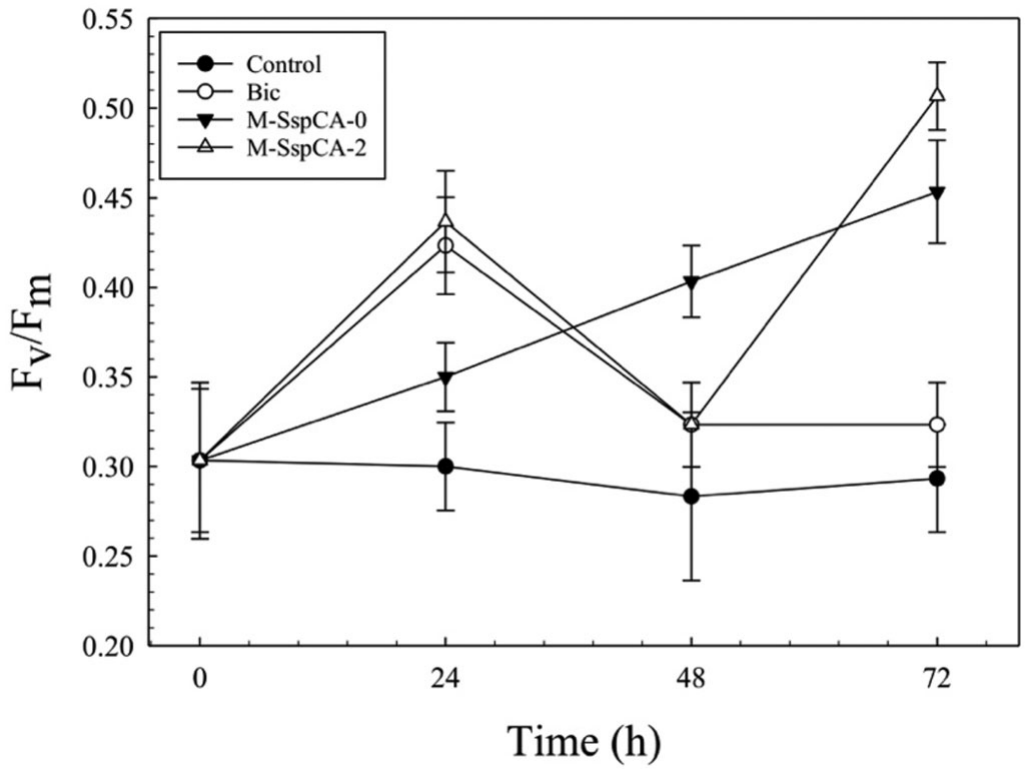
Maximum quantum yield (Fv/Fm) in *Chlorella sorokiniana* control and experimental cultures (Bic, M-SspCA-0, M-SspCA-2). Error bars represent SD (*n* = 3).

Figure 4 shows the photosynthetic efficiency (F_v_/F_m_) of *C. sorokiniana* in the cultures supplemented with bicarbonate or bicarbonate containing M-SspCA. An increase of F_v_/F_m_ was observed growing *C. sorokiniana* in the presence of bicarbonate and M-SspCA. The microalgal photosynthesis efficiency reached its maximum value at 72 h in the cultures supplemented with bicarbonate plus the M-SspCA at time 0 and 48 h compared to the control. In contrast, the photosynthetic behaviour of the cells grown in the presence of only bicarbonate showed a maximum of F_v_/F_m_ at 24 h (about 0.42 ± 0.03) and, after that time, started to decrease at 0.32 ± 0.02 F_v_/F_m_. It can be hypothesised that the reduction of F_v_/F_m_ after 24 h is due to the low availability of bicarbonate in the medium caused by microalgal utilisation. This is supported by the fact that the presence of M-SspCA guarantees bicarbonate in the microalgal culture medium through the reaction catalysed by the thermostable CA, which converts CO_2_ of the medium into bicarbonate. Moreover, as shown in Figure 4, the addition of M-SspCA at 48 h determined an increment of the microalgal photosynthetic activity.

**Figure 5.**
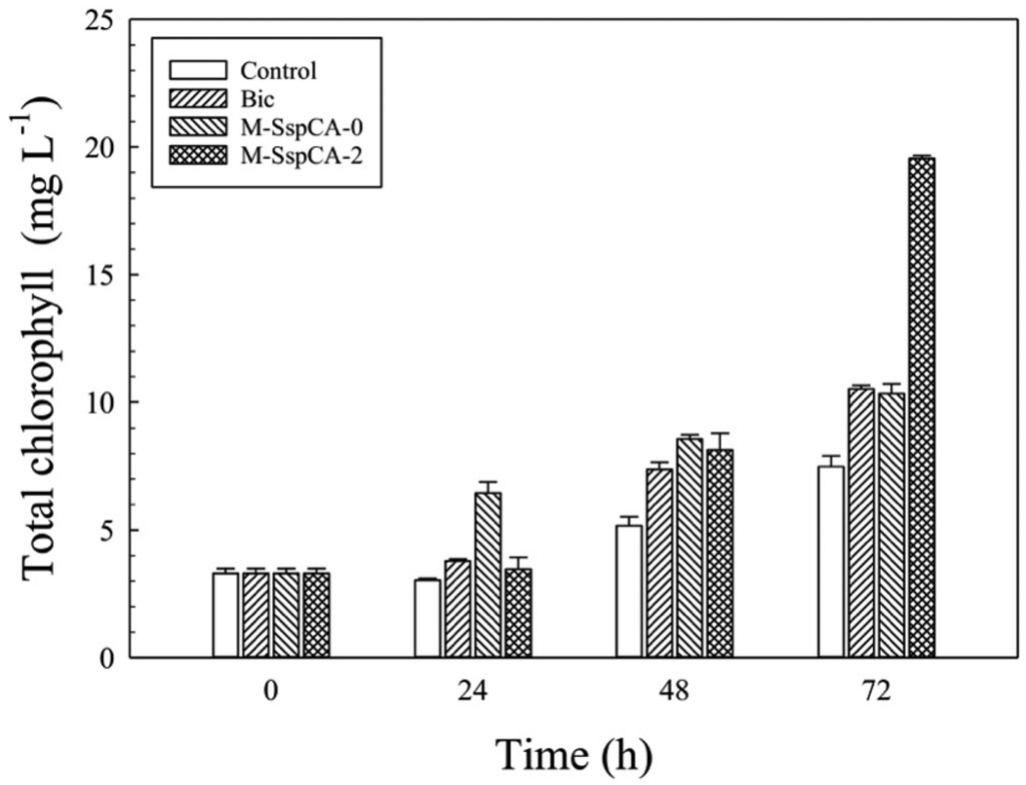
Total chlorophyll content in control *C. sorokiniana* cells and experimental cultures (Bic, M-SspCA-0, M-SspCA-2). Error bars represent SD (*n* = 3).

Furthermore, since the shortage of bicarbonate in the culture determines a condition of stress for the microalgal culture, slowing down the photosynthetic carbon fixation, the total content of the chlorophylls had been also monitored. As expected, considering the data previously shown, the photosynthetic pigment had a maximum in the microalgal culture grown in the medium containing bicarbonate and M-SspCA (Figure 5). In fact, the addition of M-SspCA, with its hydratase activity and using the CO_2_ present in the medium solution, regenerates the bicarbonate, which is necessary for increasing the microalgal metabolism and, thus, the photosynthetic pigment. The addition of M-SspCA at T0 is only responsible of an increase of the total chlorophyll contents at 24 h (Figure 5).

### 3.4. Carotenoids production

**Figure 6.**
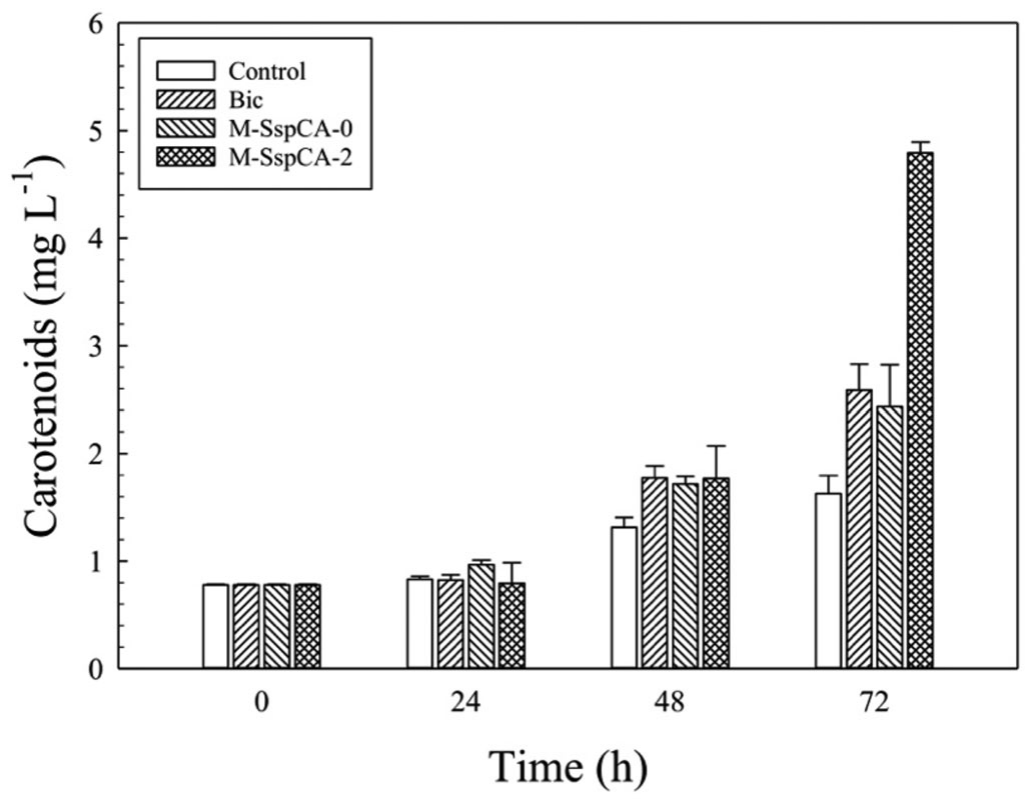
Total carotenoids content in control *C. sorokiniana* cells and experimental cultures (Bic, M-SspCA-0, M-SspCA-2). Error bars represent SD (*n* = 3).

Microalgae are considered to be one of the best commercial sources of natural carotenoids, such as β-carotene, astaxanthin, canthaxanthin, lutein, etc. These molecules are produced in a variable amount depending on microalgal growth conditions^69^. Besides, carotenoids are of great interest to humans since they are used as antioxidants, anti-inflammatories, antidiabetics, anti-obesities, antitumoral, and it has been reported that they possess a cardiovascular and neuronal protective role^69^. For this reason, we explored the carotenoids production by *C. sorokiniana* in the medium supplemented with bicarbonate and bicarbonate with M-SspCA. Figure 6 evidences the content of the total carotenoids in the *Chlorella* cells. The carotenoids content remained almost constant up to 24 h in the four conditions. At 48 h, the microalgal carotenoid production was slightly enhanced by the presence of bicarbonate or bicarbonate with M-SspCA, however, at 72 h, its amount in M-SspCA-2 became three times higher (4.8 mg L^−1^ ± 0.09) compared to the control (1.62 mg L^−1^ ± 0.17). These results are fascinating for a biotechnological application because the membrane-bound SspCA can be used in the microalgal culture for obtaining a very high production of the carotenoids.

### 3.5. Determination of the microalgal endogenous hydratase activity at 72 h

As described above, the basal specific activities of the microalgal endogenous CAs resulted to be of 20 ± 0.7 WAU/mg. Since the CA is a crucial enzyme in the carbon concentration mechanism by enhancing the conversion between CO_2_ and bicarbonate ions, the microalgal CA activity of the microalgal cells coming from the control medium has been measured at 72 h, as well as in the medium supplemented with bicarbonate and the culture medium containing bicarbonate and M-SspCA added at the two different times (0 and 48 h). The cells were extensively washed and recovered by centrifugation. To avoid the interference of the external M-SspCA added to the culture medium, a control containing M-SspCA-0 or M-SspCA-2 without microalgal cells was prepared. As a result, the CA specific activity at 72 h of the microalgal culture was of 60 ± 0.8 WAU/mg when the M-SspCA was added at 48 h to the medium culture supplemented with bicarbonate. At the same time, it resulted to be 40 ± 0.7 WAU/mg when added at time 0 and in the medium containing only bicarbonate. The addition of M-SspCA at 48 h to the microalgal medium drastically influences the metabolic efficiency of the microalgal culture, determining an increase of the photosynthetic efficiency, microalgal cell size, total chlorophyll, as well as a higher production of carotenoids (see Figures 3–6). It can be speculated that the extra-CA activity provided by SspCA may enhance the conversion of bicarbonate to CO_2_ in the proximity of the RuBisCO enzyme affecting thus the metabolic efficiency of *C. sorokiniana*.

## 4. Conclusions

In the present paper, fragments of the bacterial membranes containing a thermostable SspCA (M-SspCA) immobilised on their surface were used for exploring the effect on growth and photosynthetic efficiency of the freshwater green microalga *Chlorella sorokiniana*. M-SspCA was added to the microalgal culture medium supplemented with bicarbonate at two different times (0 and 48 h). The microalgal metabolic efficiency was investigated following the variation in the number of cells, photosynthetic activity, carotenoids production, and CA activity. The results presented in the present paper evidenced that the maximum algal growth was reached when M-SspCA is added to the culture at 48 h. The addition of M-SspCA at T_0_ mainly affects the microalgal growth and the photosynthetic efficiency at 48 h. In conclusion, it can be speculated that when the bicarbonate is consumed by the algal uptake and there is an accumulation of CO_2_ into the microalgal medium, the addition of M-SspCA at T_2_, immediately converts the CO_2_ into bicarbonate. Mainly, its effect is to increase the microalgal metabolic efficiency significantly with respect to the control medium or the medium supplemented only with bicarbonate. This is of a great interest from a biotechnological viewpoint because the freshwater green microalga *Chlorella sorokiniana* can be useful in many fields, such as photosynthesis research, human pharmaceutical production, aquaculture foods, and wastewater treatment as well as third-generation biofuels feedstock^81^.
